# Neuromodulatory effects of repetitive transcranial magnetic stimulation on neural plasticity and motor functions in rats with an incomplete spinal cord injury: A preliminary study

**DOI:** 10.1371/journal.pone.0252965

**Published:** 2021-06-04

**Authors:** Siti Ainun Marufa, Tsung-Hsun Hsieh, Jian-Chiun Liou, Hsin-Yung Chen, Chih-Wei Peng

**Affiliations:** 1 Graduate Institute of Biomedical Materials and Tissue Engineering, College of Biomedical Engineering, Taipei Medical University, Taipei, Taiwan; 2 Physical Therapy Department, Faculty of Health Science, University of Muhammadiyah Malang, Indonesia; 3 School of Physical Therapy and Graduate Institute of Rehabilitation Science, Chang Gung University, Taoyuan, Taiwan; 4 Neuroscience Research Center, Chang Gung Memorial Hospital, Taoyuan, Taiwan; 5 Healthy Aging Research Center, Chang Gung University, Taoyuan, Taiwan; 6 School of Biomedical Engineering, College of Biomedical Engineering, Taipei Medical University, Taipei, Taiwan; 7 Department of Occupational Therapy and Graduate Institute of Behavioral Sciences, College of Medicine, Chang Gung University, Taoyuan, Taiwan; 8 International PhD Program in Biomedical Engineering, College of Biomedical Engineering, Taipei Medical University, Taipei, Taiwan; 9 School of Gerontology Health Management, College of Nursing, Taipei Medical University, Taipei, Taiwan; Universita degli Studi di Catania, ITALY

## Abstract

We investigated the effects of intermittent theta-burst stimulation (iTBS) on locomotor function, motor plasticity, and axonal regeneration in an animal model of incomplete spinal cord injury (SCI). Aneurysm clips with different compression forces were applied extradurally around the spinal cord at T10. Motor plasticity was evaluated by examining the motor evoked potentials (MEPs). Long-term iTBS treatment was given at the post-SCI 5th week and continued for 2 weeks (5 consecutive days/week). Time-course changes in locomotor function and the axonal regeneration level were measured by the Basso Beattie Bresnahan (BBB) scale, and growth-associated protein (GAP)-43 expression was detected in brain and spinal cord tissues. iTBS-induced potentiation was reduced at post-1-week SCI lesion and had recovered by 4 weeks post-SCI lesion, except in the severe group. Multiple sessions of iTBS treatment enhanced the motor plasticity in all SCI rats. The locomotor function revealed no significant changes between pre- and post-iTBS treatment in SCI rats. The GAP-43 expression level in the spinal cord increased following 2 weeks of iTBS treatment compared to the sham-treatment group. This preclinical model may provide a translational platform to further investigate therapeutic mechanisms of transcranial magnetic stimulation and enhance the possibility of the potential use of TMS with the iTBS scheme for treating SCIs.

## Introduction

Spinal cord injuries (SCIs) lead to changes in motor, sensory, and autonomic functions of the spinal cord [1]. The effects of an SCI vary depending on the segment of the spinal cord that is affected [2]. The severity of the primary injury determines the grade of the patient’s neurologic state and is used as a prognostic tool [3]. In the initial stage (a few seconds to a minute) after spinal damage occurs, termed the acute phase, the primary injury mechanism leads to physiological alterations, including hemorrhaging, spinal shock, systemic hypotension, cell death, reduced blood flow, edema, and neurotransmitter accumulation [4]. In the secondary stage (minutes to weeks) after the spinal cord is damaged, termed the sub-acute phase, necrotic cell death, edema, electrolyte shifts, free-radical production, delayed calcium influx, and apoptosis continue to occur [5]. In the last stage (months to years) after spinal cord damage, termed the chronic phase, apoptosis, demyelination, glial scar formation, and alteration of neuronal circuits continue to occur [4, 5]. Moreover, cortical areas are invaded which causes axonal disintegration and reductions in the dendritic spine density and angiogenesis, and thus, information is not conveyed along the central pathway [6].

Most SCI patients experience spasticity, muscle atrophy, and urinary infections, and more than 80% have neuropathic pain [7]. Thoracic SCIs comprise the largest percentage of SCI incidences in the United States [8]. Following a thoracic SCI, paralysis of the lower limbs is the most common symptom [9]. Various rehabilitative approaches have been developed to accelerate the recovery of locomotor function, such as shift training, walking training, and balance exercises. However, those training protocols often cause injuries to SCI patients as a result of the great efforts they must exert during exercise, and there is no fully therapeutic treatment for SCI patients [2]. In the clinic, most SCIs are incomplete, rendering stimulation toward the motor cortex (M1) a strategy to reconnect the brain with the spinal cord below the lesion [10]. Neural activation activated by transcranial magnetic stimulation of the M1 is a current assessment approach to evaluate integration of the descending pathway after an SCI [10, 11].

Previous studies proved that TMS provides information of the motor status of patients with a deficit in motor function from varying causes and with different degrees of severity [12]. When TMS is applied over M1 at the appropriate intensity, the electromagnetic pulse activates excitable cells and passes through the corticospinal tract (CST) and the peripheral nerves to muscles [13, 14]. The excitability of M1 and conduction through the CST, termed motor-evoked potentials (MEPs), can be recorded with electromyographic (EMG) surface electrodes [15]. MEP recordings also provide objective information for assessing central motor pathways and monitoring central motor deficits [13, 15]. Moreover, the MEP amplitude shows the integrity of neuronal axons from M1 to spinal motoneurons and have been used in different neurology deficit at subclinical level [14]. An abnormal MEP can be found in patients with any level of corticospinal dysfunction, while the presence of MEP shows the integrity of the pyramidal tract [16]. Importantly, TMS has a role in determining the value of pyramidal tract dysfunction, especially in motor deficit cases, whereas the role of the MEP is unequivocal when there are associated motor deficits [13]. For instance, after spinal cord damage, white matter loss is believed to lead to the loss of neurological function among SCI patients [17]. Furthermore, a good correlation was seen between the MEP size and motor ability in SCI patients, as well as between the MEP size and radiological lesions in stroke and cervical myelopathy patients [14, 18].

Furthermore, TMS is an accepted approach for neuromodulation and neurostimulation of the central nervous system (CNS) with beneficial effects reported in various neurologic disorders [19–23]. Repetitive (r)TMS induces a neuro-suppressive effect with a lower frequency (below 1 Hz) and induces a neurogenic excitatory effect with a higher frequency (above 5 Hz) [24]. Previous studies showed that different rTMS frequencies can be used to reduce spasticity and neuropathic pain [25–30]. Intermittent theta-burst stimulation (iTBS) is a TMS protocol that can induce long-term potentiation (LTP) like plasticity at M1 with a few minutes’ stimulations leading to 60 min of facilitated cortical excitability changes [31, 32]. However, few studies of iTBS have tried to identify therapeutic roles of rTMS in SCIs. A previous clinical study revealed that iTBS reduced the level of spasticity and altered cortical excitability in subjects with chronic incomplete SCIs [33–35]. However, relationships between changes in cortical excitability and motor behavior have yet to be predicted [34].

Different SCI severities in subjects may possibly have discrepant responses to the same rTMS rehabilitative protocols. However, few studies have reported on whether the severity of SCI conditions has a great influence on therapeutic outcomes of rTMS. Since rats have gained great popularity as primary species for investigating SCIs [36, 37] the present study developed a clip compression approach that can produce hindlimb lesions in an SCI rat model with mild, moderate, and severe severities. We utilized this technique because compression affects the white matter of the spinal cord similar to what occurs in clinical SCIs. In the current study, SCI rats of all three severities underwent 2 weeks of an rTMS rehabilitative intervention. To epidurally activate M1, we used iTBS, which was demonstrated to facilitate cortical excitability [11]. We examined changes in cortical excitability represented by MEPs in hindlimb muscles. Motor function and biochemical tests were also conducted at multiple time points. These results may contribute to our understanding of the influence of the severity of SCI conditions on outcomes of neural plasticity and motor function with rTMS treatment and can serve as a basis for developing effective strategies for valuable future rehabilitative interventions.

## Materials and methods

### Animal preparation

**Fig 1:**
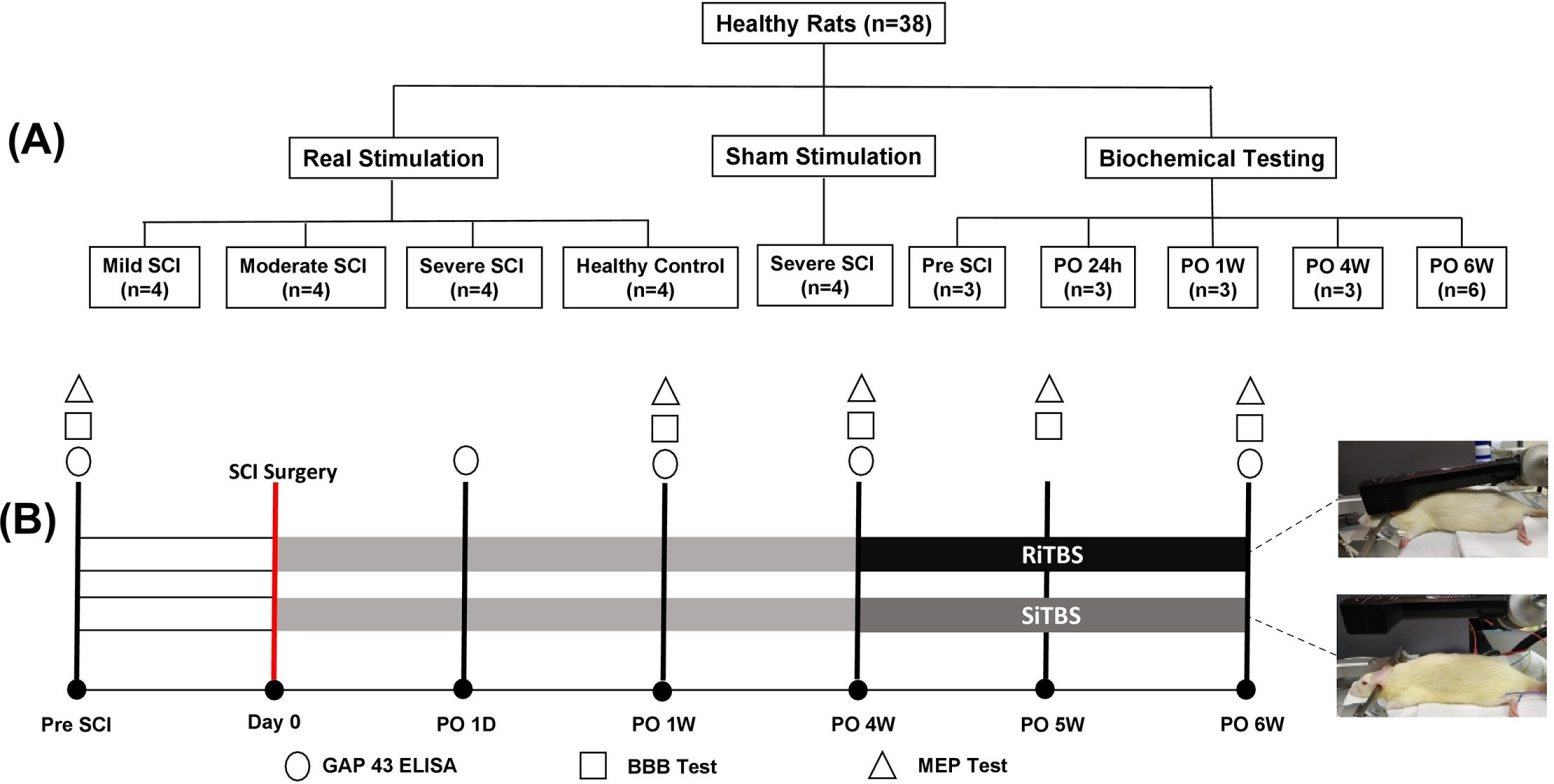
Schematic of the experimental design in this study. (A) Time-course analyses of motor-evoked potentials (MEPs), the Basso Beattie Bresnahan (BBB) scale, and a growth-associated protein (GAP)-43 ELISA were conducted. (B) BBB and MEP testing were performed one time pre-spinal cord injury (SCI) and once each at weeks 1, 4, and 6 following the SCI. A GAP-43 ELISA was performed pre-SCI and post-SCI at 24 h, and at weeks 1, 4, and 6 following a severe SCI and after intermittent theta burst stimulation (iTBS). *PO, postoperative; D, day; W, week; RiTBS, real intermittent theta-burst stimulation; SiTBS, sham intermittent theta-burst stimulation. *PO, postoperative; W, week; RiTBS, real intermittent theta-burst stimulation; SiTBS, sham intermittent theta-burst stimulation.

All experimental protocols involving the use of animals in this study were approved by the Institutional Animal Care and Use Committee (IACUC) at Taipei Medical University (TMU) with approval number (LAC-2018-0402), in accordance with the relevant *Guide for the Care and Use of Laboratory Animals* published by the National Institutes of Health. All animal experiments in this study were carried out in compliance with Animal Research: Reporting of In Vivo Experiments (ARRIVE) guidelines. Thirty-eight adult male Sprague-Dawley rats (BioLASCO, Taipei, Taiwan) weighing 276~300 g were utilized in the present study. Among them, 20 rats were randomly divided into actual stimulation (separated into healthy controls, mild-SCI, moderate-SCI, and severe-SCI; *n* = 4 in each group; Fig 5A) and sham stimulation (severe-SCI; *n* = 4; Fig 1A). The remaining 18 rats were used for biochemical testing. All rats were separated into four equal groups at pre-SCI, and at 24 h, and 1 and 4 weeks after creating a severe SCI; in addition, two groups underwent either actual or sham stimulation at 6 weeks after a severe SCI (*n* = 3 in each group; Fig 1A). The experimental scheme is illustrated in Fig 1B. Rats were housed in a temperature- and humidity-controlled room (21~23°C) with a 12-h light/dark cycle and ad libitum access to food and water at the animal center of TMU before the experimental procedures.

### SCI surgery

A compression injury in the SCI group was produced using commercial clamping clips (Micro Vascular Clip; RS-6470, RS-6472, RS-6474; Roboz Surgical Instrument, Gaithersburg, MD, USA). A rat was deeply anesthetized with an intraperitoneal (i.p.) injection of tiletamine-zolazepam (50 mg/kg, i.p.; zoletil, Vibac, Carros, France) and xylazine (10 mg/kg, rompun, Bayer, Leverkusen, Germany). A dorsal laminectomy was performed at T10 to expose the spinal cord, and three different compression clips producing forces of 35, 40, and 60 g were individually applied for 60 s using an applicator (Micro Clip Setting Forceps; RS 6496; Roboz Surgical Store, MD, USA) to produce injuries of varying severities, i.e., mild, moderate, and severe [37, 38]. All surgical procedures were performed under aseptic conditions. Rats were monitored daily, and postoperative care was administered. Each rat’s bladder was squeezed twice daily until spontaneous voiding occurred, and to prevent infection, an ampicillin (100 mg/kg, subcutaneously (s.c.), Novopharm, Toronto, Canada) injection was administered intramuscularly for 5 days [38].

### MEP assessment

**Fig 2:**
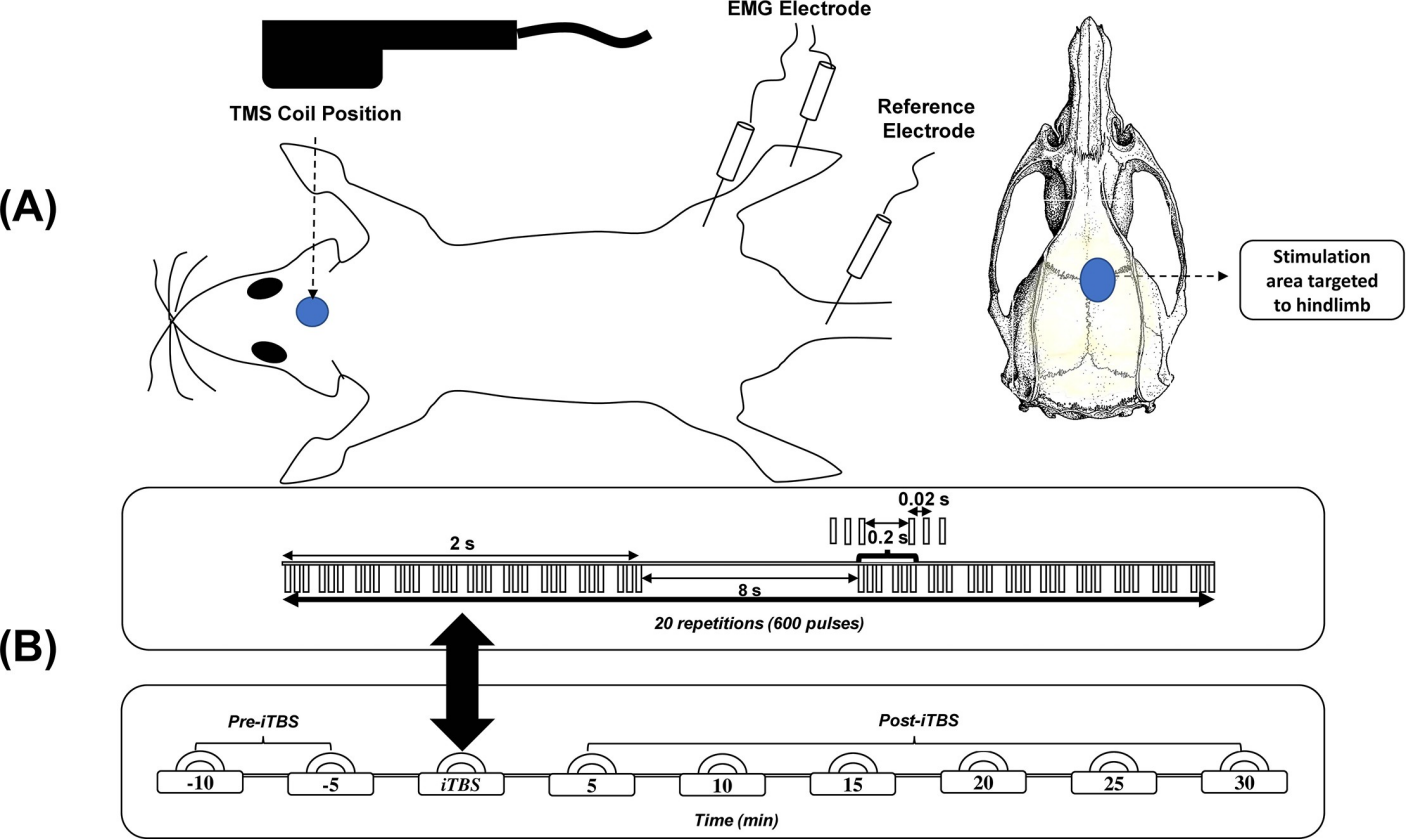
Protocol for motor-evoked potential (MEP) measurements. Placement of the transcranial magnetic stimulation coil and electrodes to record MEPs from rat models (A). Intermittent theta-burst stimulation (iTBS) consisted of 600 pulses that burst within 192 s; 15 MEPs were recorded every 5 min twice before iTBS and six times after iTBS (B).

A rat was placed in a stereotaxic apparatus after i.p. anesthetization with tiletamine-zolazepam (50 mg/kg, i.p.; zoletil) and xylazine (10 mg/kg, rompun) [39]. Monopolar uninsulated 27G stainless steel needle electrodes (Axon Systems, Hauppauge, NY, USA) were inserted unilaterally into the bicep femoris muscle belly of the hindlimb to record EMG activity. The reference electrode was inserted into a paw, while the ground electrode was inserted into the tail [40]. Signals were recorded with a Biopac MP-36R four-channel system (Biopac System, Goleta, CA, USA). All TMS protocols were performed using a MagVenture MagPro and Cool-40 Rat circular coil (Tonica Electronic, Farum, Denmark). The circular coil was fixed such that it was in slight contact with the scalp surface to elicit MEPs to the hindlimb (locator coordinate AP: ±1.0 mm; ML: ±1.25 mm; bregma as the center) [41]. A single-pulse of TMS evoked MEPs to evaluate cortical excitability after a session in SCI rat models. The minimal intensity of TMSs required to elicit MEPs from the bicep femoris muscle with at least a 20-μV amplitude in five of ten consecutive trials during muscle relaxation caused by anesthesia was termed the resting motor threshold (RMT) [40]. The intensity of TMS for the RMT was documented to be 100% of the machine output percentage which is associated with the maximal strength of the magnetic field [40]. The MEP amplitude was recorded every 5 min twice pre-iTBS as the baseline condition and six times post-iTBS (Fig 2B). The recorded MEP consisted of 15 single pulses at 10-s intervals at 120% RMT. Peak-to-peak amplitudes were analyzed offline.

### iTBS protocol

iTBS used in this study consisted of three-pulse bursts at 50 Hz repeated at 5 Hz. A 2-s train of TBS was repeated every 10 s for 20 repetitions, for 600 pulses in total (Fig 2A) [31]. An 80% RMT was used as the iTBS intensity [39]. iTBS was administered for 2 weeks at postoperatively (PO) 5~6 weeks, for 5 consecutive days each week, 10 times in total (Fig 2B). The iTBS protocol for the sham stimulation group was the same as that for the actual stimulation group, except the coil was placed 8 cm above the rat’s head [42].

### Basso Beattie Bresnahan (BBB) scoring

The BBB scale is a locomotor scale to assess a rat’s hindlimb function, consisting of 22 points (normal score, 21; no hindlimb function, 0), and categorized into early (0~7), intermediate (8~14), and late stages (15~21) [43]. We placed a rat into the track area we designed to monitor hindlimb function for 5 min. We used a screening scoring sheet developed by Basso to reduce subjectivity during the assessment [37, 38, 43, 44].

### Biochemical testing: GAP-43 enzyme-linked immunosorbent assay (ELISA)

The brain and spinal cord from severe-SCI groups were harvested at PO 24 h, and at 4 and 6 weeks in both the actual and sham stimulation groups to check GAP-43 expression, which represents axonal regeneration. Tissues were homogenized in phosphate-buffered saline (PBS; 1:9) on ice, then centrifuged for 5 min at 5000 ×*g* to obtain the supernatant. Analyses of GAP-43 levels in tissue samples were carried out in duplicate wells with an ELISA kit (MyBioSource, San Diego, CA, USA). The standard working solution and a sample were added side by side into a well (100 μl/well) and incubated for 90 min at 37°C. A biotinylated detection antibody (Ab) working solution at 100 μl/well was added after removing the liquid from each well, and further incubated for 1 h at 37°C. Wash buffer (350 μl) was added to each well three times. A horseradish peroxidase (HRP)-conjugated working solution (100 μl) was added to each well and incubated for 30 min at 37°C. Wells were washed five times with wash buffer, and 90 μl of substrate reagent was added. The plate was protected from light and incubated for 15 min at 37°C. Stop solution (50 μl) was added to each well before determining the optical density (OD) with a microplate reader set to 450 nm. Sample values are expressed as ng/ml and were calculated from the standard curve of the GAP-43 antigen.

### Statistical analysis

All analyses were performed using SPSS vers. 17.0 (SPSS, Chicago, IL, USA) with *p* < 0.05 considered significant. All data are presented as the average and standard error of the mean (SEM). A two-way repeated (RM)-measures analysis of variance (ANOVA) was used to analyze the iTBS effect performed on the MEP amplitude with group (healthy control, mild-SCI, moderate-SCI, severe-SCI with actual stimulation, and severe-SCI with sham stimulation) as the between-subject factor and time (5, 10, 15, 20, 25, and 30 min after iTBS) as the within-subject factor. A one-way ANOVA was used to analyze the difference in MEP amplitudes between each time point and the baseline in every group. Multiple comparisons were performed using a one-way ANOVA with a post-hoc least significant difference (LSD) test to analyze the difference in BBB scores between all groups and at each time point. Expressions of GAP-43 in the brain and spinal cord from the severe-SCI group were analyzed at different time points.

## Results

### Effects of iTBS on MEP

**Fig 3:**
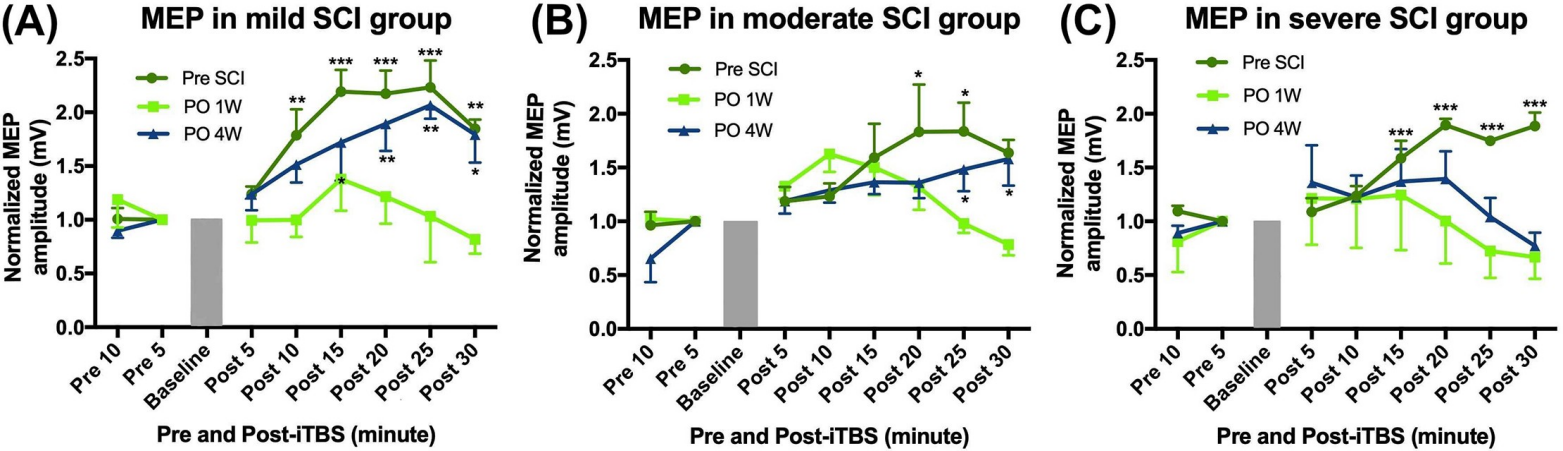
Single intermittent theta-burst stimulation (iTBS) effect on the motor-evoked potential (MEP) amplitude. Time course of average changes in the MEP amplitude from the mild-spinal cord injury (SCI) group (A), moderate-SCI group (B), and severe-SCI group (C). Each point corresponds to the mean and standard error of the MEP amplitude expressed as a ratio to the last block of baseline responses. * *p* < 0.05, ** *p* < 0.01, *** *p* < 0.001, by post-hoc Fisher’s LSD test on the MEP size: between each time point post-SCI compared to the MEP amplitude average of the last block pre-iTBS.

**Fig 4:**
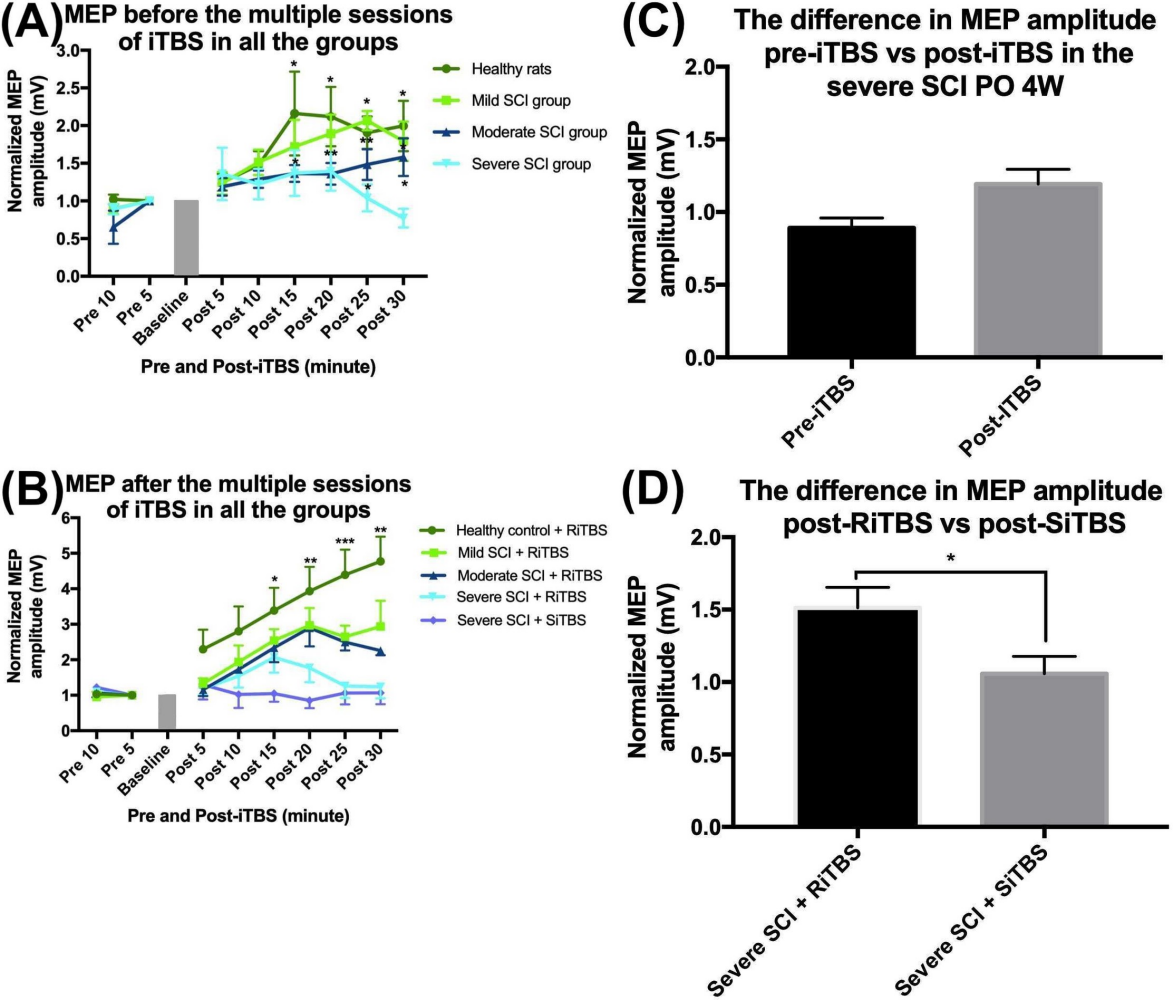
Effects of multiple sessions of intermittent theta-burst stimulation (iTBS) on the motor-evoked potential (MEP) amplitude. Before the multiple sessions of iTBS, the MEP amplitude had increased in the healthy control group and showed less facilitation in the spinal cord injury (SCI) groups (A). After multiple sessions of iTBS, the MEP amplitude increased in all real stimulation groups but was less facilitated in the sham stimulation group, and the effects of iTBS in the five groups significantly differed from each other (B). There was no significant difference between MEP amplitudes pre- and post-iTBS in the severe-SCI group at postoperative 4 weeks (PO 4W) (C). There was a significant difference between the MEP amplitude in the severe-SCI group + RiTBS and severe-SCI group + SiTBS at PO 6W (D). * *p* < 0.05, ** *p* < 0.01, *** *p* < 0.001, by post-hoc Fisher’s LSD test on the MEP size between each time point post-SCI compared to the baseline.

Fig 3 shows that there was a significant enhancement in the MEP amplitude at every time point post-SCI compared to the pre-SCI baseline. Following an SCI, there was a change in the MEP amplitude at all three different severities (mild, moderate, and severe). A two-way RM ANOVA confirmed significant changes in the MEP amplitude at pre-SCI, PO 1W, and PO 4W with the factor time (*F*_7,63_ = 7.70, *p* < 0.001 in the mild-SCI group; *F*_7,63_ = 10.17, *p* < 0.001 in the moderate-SCI group; and *F*_7,63_ = 3.33, *p* = 0.004 in the severe-SCI group) and group factor (*F*_2,9_ = 14.81, *p* = 0.001 in the mild-SCI group but was not significant in either the moderate-SCI group *F*_2,9_ = 0.64, *p* = 0.55 or severe-SCI group *F*_2,9_ = 1.51, *p* = 0.27). Fig 4 shows differences in the MEP amplitude among all groups at PO 4W and 6W. A two-way RM ANOVA showed that there was no significant difference between each group at PO 4W before the multiple sessions of iTBS (*F*_3,12_ = 2.89, *p* = 0.08). A two-way RM ANOVA at PO 6W revealed significant differences in MEP amplitudes among the five groups with the factors time (*F*_7,105_ = 23.61, *p* < 0.001) and group (*F*_4,15_ = 6.96, *p* = 0.002). At PO 6W, a one-way ANOVA confirmed a significant difference in MEP amplitudes among all groups at each time point (*F*_4,19_ = 3.91, *p* = 0.02 at 15 min; *F*_4,19_ = 5.96, *p* = 0.004 at 20 min; *F*_4,19_ = 10.07, *p* < 0.001 at 25 min; and *F*_4,19_ = 9.11, *p* = 0.001 at 30 min). There was no significant difference revealed by an independent *t*-test between pre- and post-iTBS in the severe-SCI group at PO 4W (*p* = 0.24). Nevertheless, an independent *t*-test also revealed a significant difference between severe-SCI + RiTBS and severe-SCI + SiTBS at PO 6W (*p* = 0.02).

### BBB score following an SCI and iTBS

**Fig 5:**
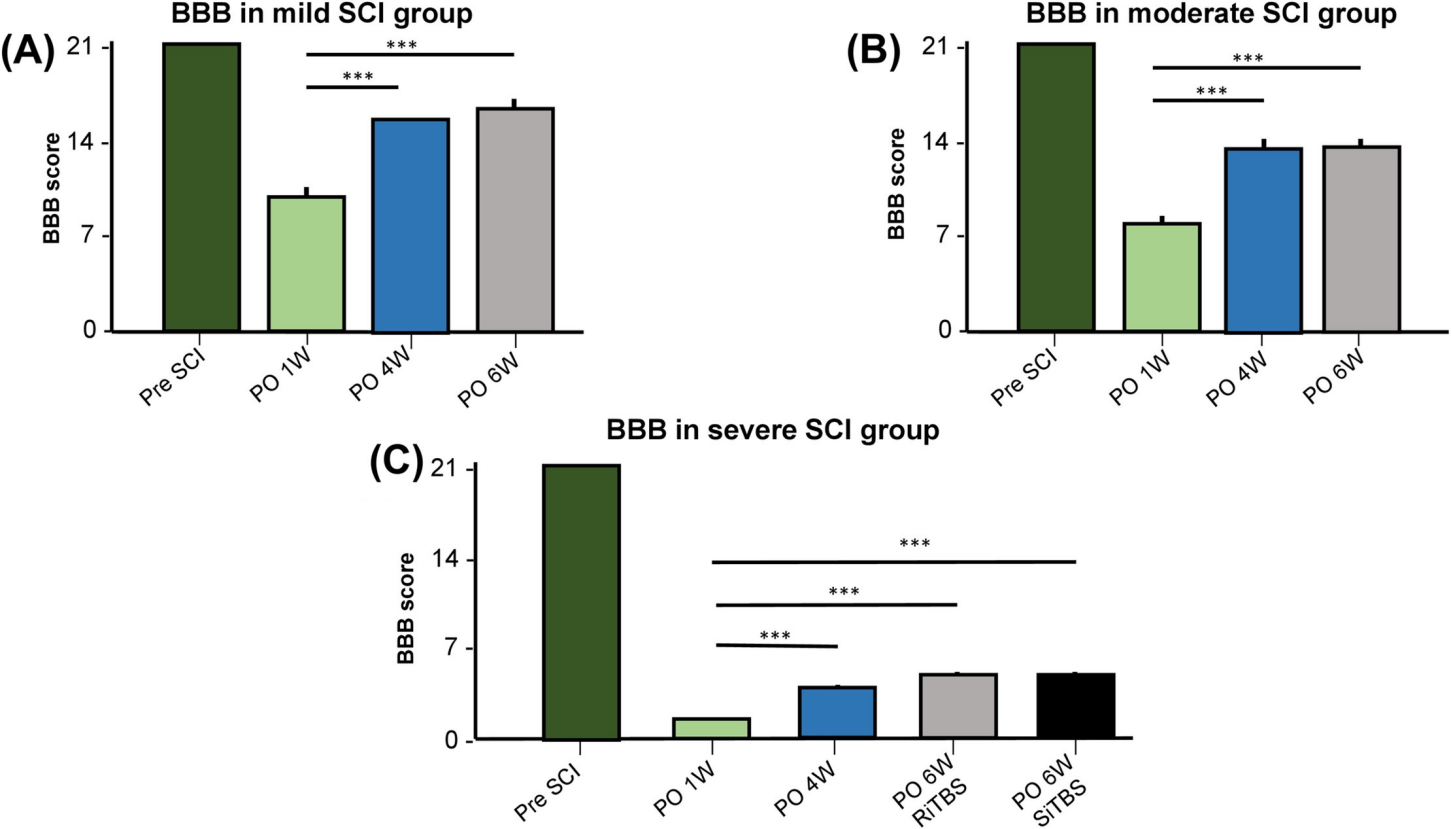
Time course of average changes in Basso Beattie Bresnahan (BBB) scores. In rats with mild (A), moderate (B), and severe spinal cord injury (SCI) (C) and intermittent theta-burst stimulation (iTBS) treatment. *** *p* < 0.001, by post-hoc LSD multiple-comparison tests on the locomotor function between different points.

Fig 5 presents significant changes in the BBB score pre- and post-SCI at different time points. A one-way ANOVA with a post-hoc LSD test confirmed significant changes in the BBB score post-SCI compared to pre-SCI represented by time (*F*_2,11_ = 467.18, *p* < 0.001 in the mild-SCI group; *F*_2,11_ = 944.14, *p* < 0.001 in the moderate-SCI group; and *F*_2,11_ = 2523.5, *p* < 0.001 in the severe-SCI group). The BBB score was significantly enhanced after RiTBS at PO 6W in the healthy control group, mild-SCI group, and moderate-SCI group compared to the severe-SCI group + SiTBS (*p* < 0.001). Nonetheless, there was no significant difference in the BBB score between the severe-SCI group stimulated by RiTBS or SiTBS.

### Effects of iTBS on GAP-43 expression

**Fig 6:**
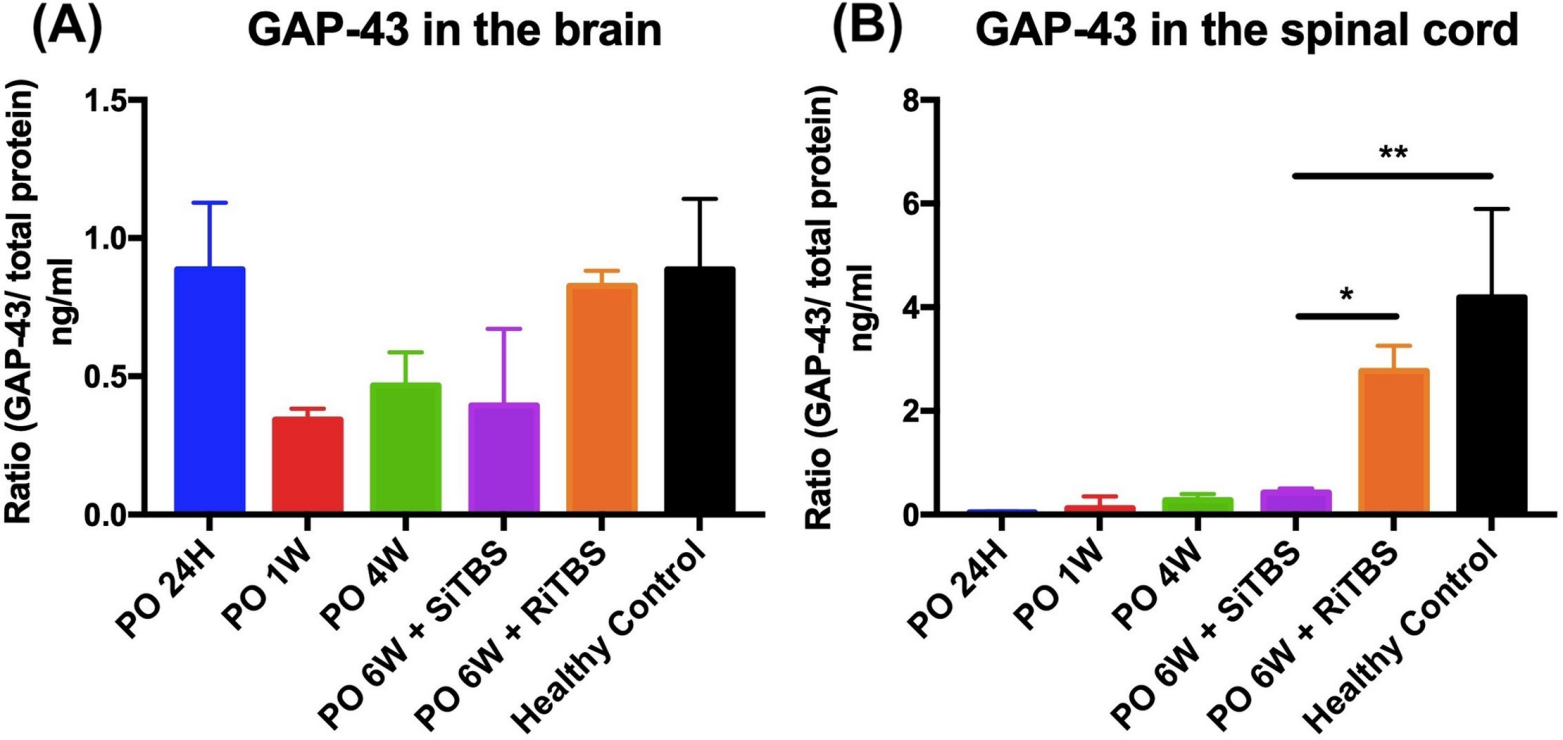
Time course of average changes in expressions of growth-associated protein (GAP)-43. GAP-43 levels in the brain (A and spinal cord (B) at different time points. Each different time point in both the brain and spinal cord corresponded to the ratio of GAP-43/total protein. * *p* < 0.05, ** *p* < 0.01, by post-hoc LSD multiple-comparison tests on GAP-43 expression from each time point compared to the severe-spinal cord injury (SCI) + sham intermittent theta-burst stimulation (SiTBS) group.

Fig 6 presents GAP-43 expressions in tissues (brain and spinal cord) at six different time points (pre-SCI, PO 24H, PO 1W, PO 4W, and PO 6W) between the real and sham stimulation groups following a severe SCI. GAP-43 was detectable at every time point we checked. Yet, a one-way ANOVA with the post-hoc LSD test confirmed no significant difference in GAP-43 expression in brain tissues among all groups (*F*_5,17_ = 1.82, *p* = 0.18). In spinal cord tissues, there was a significant difference among all groups (*F*_5,17_ = 5.71, *p* = 0.006). Further, multiple comparisons revealed there was a significant difference in the severe-SCI + SiTBS group compared to the severe-SCI + RiTBS group (*p* = 0.043) and compared to the healthy controls (*p* = 0.003).

## Discussion

To our best knowledge, this is the first study to investigate the effects of an rTMS intervention on modulating neural plasticity and motor functions in rats with an incomplete SCI of different severities. In the present study, we successfully conducted one of the human rTMS protocols (iTBS) at three different severities in a rat model of compression SCIs. Impairment due to a compression injury of different severities was tested by a behavioral investigation. After being treated with RiTBS, the mild-SCI group had better results of motor cortex excitability compared to the moderate- and severe-SCI groups. Further, changes in GAP-43 expression in our study were closely related to neural plasticity after the injury, as proven by biochemical analyses. Neural plasticity induced by actual iTBS increased in the mild-SCI group and decreased in the most severe SCI group.

### iTBS induced changes in the MEP

The MEP elicited by TMS is used to estimate cortical excitability in both healthy and diseased conditions. Several previous studies mentioned that in addition to neuroimaging results, MEP produced by TMS can support a clinical diagnosis of SCIs [33, 45, 46]. MEP results provided information that can possibly be used to assess SCIs based on the injury severity and can estimate long-term outcomes by its application in support of the healing process post-injury [45]. Yet, no change in the MEP within a year indicated a lack of remyelination of the spared pathways [47]. Previous TMS studies evaluated the plasticity of SCI cases in clinical and animal models [33, 46]. In healthy rats, MEP can be consistently activated until 30 min after a single session of iTBS as reported in a previous study [39]. During the recording of the MEP, we anesthetized rats from all groups to prevent stress and better control them, while iTBS in a human study was applied in a conscious condition. However, we found that it was difficult to induce plasticity in conscious animals compared to unconscious animals [48, 49].

In most SCI studies, elicited MEPs were more easily monitored in an upper limb muscle than in a lower limb muscle [45, 50]. However, MEPs elicited from both upper and lower limbs have the potential to allow assessment of the SCI based on the injury severity and predict outcomes [45, 47, 51]. A single MEP recording consisted of eight measurements in total, including twice at pre-iTBS and six times every 5 min until 30 min post-iTBS. A level of 120% RMT was used to evoke MEPs in the present study. Different time points and the RMT we used were the same as in previous SCI studies by TMS [10, 37, 39, 52, 53]. The iTBS intensity we used was 80% of the RMT, which was higher than the intensity given to humans. Nevertheless, the intensity we used was the same as in previous studies of animal disease models [39, 52], and it did not greatly differ from other studies of SCI rats, which used 90% RMT [11] and 75% RMT [10, 53].

iTBS is expected to increase the MEP amplitude size [31]. As a result of iTBS treatment in our study, we elicited an MEP amplitude of no less than 20 μV, which indicated that there was a facilitative effect of iTBS not only in healthy controls but also in all SCI groups. Contrary to previous SCI animal models, MEP was elicited and showed a facilitative effect of TMS only in healthy rats but was absent from injured rats [9]. Our MEP results from all SCI groups showed a smaller facilitative effect of iTBS at PO 1W which gradually improved at PO 4W and 6W. However, our results revealed that the severe-SCI group had a smaller facilitative effect of iTBS-induced plasticity compared to the mild- and moderate-SCI groups. From the models in our study, we revealed that motor plasticity was reduced in SCI rats, especially in the early stage post-injury.

MEP responses are highly correlated with cortical excitability and the nerve root condition in disease models [19, 47]. However, iTBS did not alter the MEP amplitude in several diseases, including multiple-system atrophy [54], Parkinson’s disease [55], and Tourette’s syndrome [56]. One previous study revealed that single iTBS treatment in SCI patients increased the MEP in 25% of participants and decreased the MEP in 50% of participants [34]. In another study, 52% of SCI participants showed an increase in the MEP amplitude, and 48% of participants showed suppression of the MEP amplitude [57]. Those previous results revealed an unclear direction between the MEP amplitude and motor performance [34]. Yet, the SCIs in our animal study showed a contrary outcome. There was facilitation after iTBS treatment in motor cortex activation that could be maintained for at least 30 min after stimulation [10, 11, 39, 53]. Due to a lack of SCI studies eliciting MEPs from a hindlimb muscle, our results indicated that iTBS is a promising therapeutic strategy with non-invasive administration, and with a short duration in a single session, it can elicit a motor response from a hindlimb muscle in SCI models.

### iTBS induced locomotor function following an SCI

The reduction in the MEP amplitude size was also correlated with spasticity as one of the most common signs in humans with an SCI [58]. Spasticity causes muscle spasms, and increases muscle tone, hyperreflexia, and involuntary movements. However, the mechanism is still not completely understood. In patients with an incomplete SCI and spastic muscles, the contribution of the corticospinal tract was demonstrated by TMS. Results showed that smaller MEP amplitude sizes over the area of M1 were found in patients with higher spasticity compared to healthy controls and patients with low spasticity [58]. A previous study showed that a high frequency of rTMS could effectively decrease spasticity in patients with multiple sclerosis and stroke [59]. In patients with a chronic incomplete SCI, a high frequency of rTMS was also demonstrated, and the spasticity significantly declined after 1 week of stimulation [25, 26]. In parallel, the spinal cord pathway is believed to mediate the neuronal mechanism of spasticity, and TMS is a viable strategy for decreasing spasticity after an SCI [60].

For ethical reasons, cervical injury needs to avoid preventing the loss of locomotor function of both the forelimbs and hindlimbs [36]. We chose T10 as the affected level to observe loss and recovery of hindlimb function. Previous SCI studies had difficulties restoring the function of the lower limbs in both humans and animals. BBB scores are used to assess the performance of three joints (knee, hip, and ankle) following thoracic injury [61]. BBB might not be applicable to determine the locomotor function for an injury at the thoracic level. One study that created a contusion injury in the lumbosacral region revealed that no behavioral changes could be assessed by BBB scores [62]. Further, the BBB score focuses on joint movement, weight-bearing, coordination between limbs, postural view, and the positions of the tail and feet [43, 61].

In accordance with our study, at pre- and post-RiTBS times, the healthy control group in our study had similar BBB scores, while mild- and moderate-SCI groups exhibited a decrease in the BBB score at 1 day after surgery. A decrease in the BBB score indicates poor mobility due to paralysis. However, BBB scores had increased at PO 3W in the mild-SCI group and at PO 4W in the moderate-SCI group indicating improved coordination of the forelimbs and hindlimbs. The severe-SCI group in our study did not pass the intermediate-phase or even the late-phase, the same as in a previous study [61]. The hindlimb muscle was flaccid 1 week after SCI surgery and improved to being spastic as time went by. The average score in that previous study was never more than 5 until 12 weeks after surgery. In our study, there was no significant enhancement of the BBB score in any SCI group at PO 6W. This result revealed that there was no meaningful iTBS effect in improving locomotor function following an SCI. Long-term stimulation sessions may produce better results in locomotor function.

### iTBS induced GAP-43 expression

The role of GAP-43 in the brain of animal models, especially rodents, has been studied and is involved in synapse formation [63]. Yet, the proper function of GAP-43 is still unclear. However, evidence from several studies showed that GAP-43 is highly expressed in mature axons during regeneration [64, 65]. When GAP-43 is upregulated, it has a high potential to promote reinnervation and nerve sprouting from a lesioned area [66]. In contrast, downregulation of GAP-43 in disease models in both brain tissues and cerebrospinal fluid (CSF) showed that nerve growth was delayed [67]. High levels of GAP-43 are found in the limbic system, specifically in synaptic-contact areas [68–70]. Brain tissues are one such area, while in certain areas, such as in somatosensory, motor control, and peripheral organs, it is essentially absent [70].

In the present study, we checked GAP-43 expression in brain and spinal cord tissues. As a result, GAP-43 was detected, and there were some changes in its expression depending on the time point related to the recovery process. There were no significant changes in GAP-43 in the brain among all groups we checked, while there were significant changes of GAP-43 in the healthy control group and severe-SCI + RiTBS group compared to the severe-SCI + SiTBS group. A previous study revealed that GAP-43 was reduced in multiple sclerosis and increased when there was remyelination in the lesioned area [67]. As time went by after SCI surgery, we found enhancement of GAP-43 levels in both brain and spinal cord tissues. As a result, there was no significant difference in GAP-43 levels in spinal cord tissues between PO 6W and the healthy control group. There was a study to check GAP-43 levels in the early stage [71] and late stage of Alzheimer’s disease and Parkinson’s disease [72, 73] when GAP-43 was mainly reduced.

Known as a specific marker of axonal regeneration, GAP-43 has a close connection with axonal elongation and neural reconstruction after an injury occurs [74]. The involvement of GAP-43 in neural development and regeneration makes this protein crucial for synaptic plasticity [75, 76]. A previous study of GAP-43 in SCI cases showed increased levels in spinal cord tissues and dorsal root ganglia of neurons after treatment with interleukin (IL)-6 in an SCI model by a subarachnoid injection [76, 77]. Those results supported our findings of the GAP-43 ELISA in which we checked all groups in our study, and actual stimulation produced higher GAP-43 expression compared to the sham-stimulation group in both brain and spinal cord tissues. Results of the present study suggest that RiTBS promotes axonal regeneration via non-invasive stimulation in SCI models. The molecular mechanism through which RiTBS exerts a beneficial effect may be attributed to upregulation of GAP-43 expression, and this provides a new vision for developing TMS protocols as new therapeutic strategies for SCI.

### Clinical implications

Our result showed iTBS significantly increased MEPs in healthy rats [39], yet some previous SCI in human study showed vary in results, probably due to lack significant and neurophysiological status where the biggest pyramidal axon has a normal conduction [78]. On the other hand, SCI rats may produce better clinical results compared to SCI patients due to the stability and ability to reduce the discrepancy [79]. For translational studies, it will be meaningful to examine the effects of TMS in animal models. Many studies have utilized animal models, especially rats to understand the mechanistic side [42]. Most of those studies focused on cellular activity, while physiologic responses, such as MEP, have rarely been studied in animal models. The use of animal models has the potential to boost our understanding of pathophysiological phenomena, thereby relying on the goal of diagnosing and treating SCIs in further studies targeting synaptic plasticity [39].

Among animal models, iTBS was sensitive enough against the intensity given during stimulation sessions. Changing the intensity can activate different circuits, not only the cortical area, and with a coil larger than the rat’s brain, it is also possible to activate other areas not only M1 [80]. As a result, long-term effects of clinical studies particularly depend on the MEP pattern [31]. Hence, TMS in animal models bridges plasticity studies between animals and humans, especially for motor pathways. In order to clarify the mechanism, enhance the protocol, and find effects of changes in intensity, protocols for humans can be manipulated and examined in animal models [57]. Animal disease models can potentially match findings with SCI in humans to establish the pathophysiological and therapeutic effects of TMS protocols [39].

Our findings showed that changes in MEPs could be used to evaluate cortical excitability in patients with motor deficit signs, the same as in a previous study [13, 46]. In healthy rats, MEPs can be easily found and increased after a single session of iTBS. Furthermore, the significant presence of MEPs can be found with mild severity levels. Since MEPs elicited by TMS can support clinical findings of any degree of severity and can potentially be used to examine motor functions before and after stimulation, they parallel previous results that TMS was correlated with a patient’s motor status with motor deficit signs from any causes and any degrees of severity [46].

Some limitations should be acknowledged. Our study created three different SCI severities, yet the small sample size in each group is the main limitation which may offer less support for the statistical analyses to find conclusive results. However, all animal models from each group underwent a surgical procedure at the same level, although with different amounts of force, and both MEP and BBB were evaluated and showed significant changes in the results. Another limitation is that we delivered a single iTBS treatment without combined therapy in one treatment session. In addition, 10 days within 2 weeks may be too short of a time to deliver non-invasive treatment for a chronic SCI. Those reasons may be considered limitations of our study and need to be improved in future studies.

## Conclusions

In the present study, we utilized three different clip forces to create SCIs of different severities in rats and conducted an iTBS protocol of an rTMS intervention for 2 weeks. Further, we tested the iTBS-induced motor plasticity of the MEP amplitude, locomotor function, and axonal regeneration. Our results showed that iTBS could potently activate cortical excitability after an SCI, and reduced iTBS-induced motor plasticity which highly corresponded with the most-severe SCI condition, especially in the early stage after the SCI. iTBS can be considered a promising SCI therapy with a short duration and non-invasive delivery. To the best of our knowledge, this is the first iTBS treatment with three different SCI severities in a rat model that focused on hindlimb recovery. Nevertheless, long-term stimulation and combined therapy might be needed to study the mechanism of iTBS in promoting motor plasticity and improving therapeutic strategies for severe SCI and other neural disorders.
